# The Halakhic Heartbeat at the Edge of Life: Navigating Maternal Brain Death and Fetal Life

**DOI:** 10.5041/RMMJ.10563

**Published:** 2026-01-28

**Authors:** John D. Loike, Rabbi Tzvi Flaum, Alan Kadish

**Affiliations:** 1 Professor of Biology and Bioethics, Touro University, New York, NY, USA; 2 Professor of Judaic Studies and Mashgiach Ruchani, Lander College for Women, Touro University, New York, NY, USA; 3 President, Touro University, New York, NY, USA

**Keywords:** Bioethics, halakhic definition of death, *pikuach nefesh*, religious status of an embryo

## Abstract

This paper presents a halakhic-ethical analysis of a 2025 case involving A.S., a brain-dead pregnant woman who was maintained on somatic support to enable fetal maturation and delivery. The case raises profound questions at the intersection of Jewish law and contemporary medical practice, particularly regarding the halakhic definition of death—brain versus cardiac cessation—and the moral status of the fetus. The paper explores divergent rabbinic opinions on whether sustaining a brain-dead body for fetal viability is halakhically permissible or obligatory. Key halakhic parameters examined include the principle of *pikuach nefesh* (saving life), the fetus as a potential *nefesh*, and the permissibility of delaying burial to perform a Cesarean section. We argue that Halakhah offers nuanced and compassionate responses to unprecedented bioethical dilemmas. Moreover, the paper affirms that Jewish law is ethically responsive, evolving through dialogue with changing human circumstances while remaining rooted in balancing reverence for life with the dignity of death. It underscores the importance of interdisciplinary collaboration between halakhic authorities and medical professionals to navigate ethically complex and medically novel scenarios with both compassion and rigor. This case illustrates that the moral courage of Halakhah’s heartbeat compels rabbinical scholars to navigate its boundaries with empathy, wisdom, and fidelity to tradition.

## INTRODUCTION

*Pikuach nefesh* (the principle of saving life) is the halakhic principle that permits violating most commandments, including killing a would-be murderer in self-defense or performing medical interventions that save a life, even if it means desecrating Shabbat or other prohibitions. The Talmud establishes the principle of *pikuach nefesh* as overriding nearly all commandments,^1^ extending even to cases involving the protection of potential life, such as a fetus beyond 40 days of gestation. Rashi states that a fetus is not a *nefesh* (a living person) as long as it has not emerged into the air of the world (i.e. been born).^2^ Ramban^3^ and Ritva^4^ apply the principle of *challel alav Shabbat achshav kidei lishmor Shabbatot harbei* (desecrate one Shabbat on his behalf, so that he may observe many future Shabbats) describing the fetus as an emerging living being that will soon be a *nefesh*.^3^

In this paper, we analyze the halakhic challenges and potential solutions for a complex case that is likely to generate diverse opinions among legal and rabbinical scholars.

## THE CASE

In early 2025, a harrowing legal and ethical drama unfolded at Emory University Hospital in Georgia, USA. A.S., a 30-year-old nurse and mother, was declared brain-dead in February 2025 following cerebral thrombosis. Doctors at Emory University Hospital decided to maintain somatic support (maintaining her respiration and circulation) because of the state’s abortion law and to allow the fetus to develop until at least 32 weeks, a stage at which fetal survival outside the womb becomes more probable. A.S.’s family did not agree to or request that she be placed on a ventilator. Instead, family members, including her mother, say they were not given a choice and were told by hospital staff they had no say, due to Georgia’s restrictive abortion law (the “Heartbeat Bill”), which they believed meant that continued life support was required for the fetus to develop. The family described the experience as “torture.”^5^ Maintaining A.S. on life support required more than merely mechanical ventilation, nutritional support, and monitoring her vitals. Physicians had to administer a regimen of hormones to maintain the pregnancy and support the fetus’s growth.

On June 13, 2025, A.S.’s son was born prematurely via emergency Cesarean section (C-section). He weighed 1 pound 13 ounces and is currently viable in the neonatal intensive care unit. On June 17, A.S. was taken off life support and buried on June 28. It is medically and halakhically significant that A.S. continued to “live” on life support for many days after her C-section procedure. This case sparked national debate and raised complex ethical questions, particularly concerning the justification for sustaining somatic support in a brain-dead pregnant woman solely to facilitate fetal viability.

This case and others like it are remarkable for many reasons. First, in the secular academic world it reignited fierce debates across legal, medical, and religious circles. American law must wrestle with fetal viability, maternal autonomy, and state-level abortion restrictions. Secondly, there is concern whether long-term life support or performing a C-section endangers the gestating fetus. It is noteworthy that a systematic review in PubMed found approximately 40 such cases in medical literature over the past several decades. In at least 28 of these cases the baby survived after delivery.^6^,^7^

## WHAT IS THE JEWISH LEGAL VIEWPOINT IN THESE CASES?

While American courts grapple with issues of fetal rights and maternal autonomy, Halakhah incorporates a distinct bioethical lens. Drawing from millennia of rabbinic thought, this paper examines several critical opinions of how Halakhah addresses maternal brain death, the legal status of the fetus, and whether life support can—and should—be continued in such cases.

Two critical halakhic parameters are examined in this paper. The first is the halakhic definition of death. Prior to the 1960s, a direct and rapid link existed between the cessation of brain function and the cessation of cardiac function. For instance, after a cardiac arrest, the brain ceased to function within minutes, and, similarly, following a severe stroke or brain trauma, the heart would stop shortly after the brain. However, modern medical technology, specifically the use of respirators, has challenged this understanding by allowing a functional heartbeat to be maintained long after the brain has ceased to function. This technological advancement raised a critical halakhic question: Does brain cessation or cardiac cessation constitute halakhic death?

The second issue discussed in this paper is the “*nefesh* status” of the embryo. Due to space, this paper does not discuss the halakhic ramifications of the following Mishnah in Arachin 1:4 that states: “A pregnant woman who is sentenced to death is not delayed from execution because of her fetus. However, if she is in active labor, the execution is delayed.” Interestingly, the first documented case in the Torah that would allow a pregnant woman to be killed with her fetus is Tamar. In addition, other halakhic issues that will not be discussed in detail are: (1) the halakhot of abortion; (2) heart transplantation; (3) the halakhic history of performing a C-section; (4) the issue of *tza’ar ha-met* (suffering of the deceased); and (5) the *hashchatat zera* (destruction of seed). What is the humanhood status of a human embryo? This is a crucial question that directly impacts whether one is permitted or even obligated to maintain this brain-dead woman on life support for months in order to deliver the baby. This paper applies these questions to resolve whether the woman can be maintained for months to allow physicians to deliver a healthy newborn.

## TWO VIEWS ON HOW HALAKHAH DEFINES DEATH

Opinion is divided on the halakhic definition of death. Rabbinical authorities such as Rabbi Moshe David Tendler zt”l,^8^ the Chief Rabbinate of Israel,^9^ and Rabbi Avraham Steinberg^10^ posit that brain death constitutes halakhic death. Rabbi Tendler, drawing on both medical expertise and halakhic rulings of Rabbi Moshe Feinstein, advocates for accepting irreversible cessation of brain activity (The Harvard Criteria of Death) as the halakhic definition of death.^11^ Rabbi Steinberg concurs, particularly when there is irreversible loss of function in the brain stem, which controls independent breathing. According to this view, a brain-dead person is legally and ritually dead, even if machines can maintain heartbeat and circulation.

Israel’s Ministry of Health recognizes brain death as a legal definition of death, and its criteria are based on neurological standards like the Harvard Criteria. However, the Israeli Respiratory-Brain Death Act (2008) expands their definition to include religious accommodations for those who view cardiac cessation as halakhic death. The definition of death under the 2008 Act^12^ can be summarized as: The time of death is defined to be either when “respiratory-brain death” (respiratory + brain criteria) is determined under the Act, or when cardiac-respiratory death is determined. Respiratory-brain death may be determined if the following conditions are met: first, the medical cause leading to cessation of brain function is known and established; and second, that there is clear clinical proof of irreversible cessation of whole-brain function (including brain-stem function) and cessation of independent respiration.

Other rabbinic authorities, such as Rabbi Mordechai Willig^13^ and Rabbi J. David Bleich,^14^ maintain that death occurs only upon the irreversible cessation of heartbeat and respiration. The physiological link of cardiac function and breathing is the critical determinant of halakhic death. In this article, we analyze this case of A.S. according to both halakhic viewpoints.

## BRAIN CESSATION AS THE HALAKHIC ENDPOINT OF LIFE

We will only cite a few reasons why some *poskim* (rabbinic authorities) favor brain cessation as the definition of halakhic death. First, there is no documented record of any person declared brain dead using what is called the Harvard Criteria who has recovered from the coma.^15^

Second, the Talmud^16^ discusses the obligation to remove the rubble of a destroyed building on Shabbat to potentially save the life of someone caught in the rubble. The sages of the Talmud ask how one establishes whether that body is alive or dead: “Until where does one check [to determine if a person is alive]?” The Talmudic response is divided; some say that one examines the victim’s nose (“until his nose”), and others say that one examines the victim “until his heart.” “One must also check the heart, for sometimes a person can die and their spirit has left them, but they are still breathing.”^17^ The Rambam,^18^ in codifying this Halakhah, states:

If a person is found under a pile of debris [on the Shabbat], they clear the debris from above him, even if there is doubt whether he is alive or dead, or whether he is a Jew or a non-Jew. They check him up until his nose. If he is not breathing, they leave him, as he is presumed dead.^18^

What is fascinating is that the Rambam omits the heart criteria in his legal rulings. The Kesef Mishneh in his commentary points out that Rambam’s exclusion of the heart from the criteria for life is a direct application of this Talmudic principle.^19^

We would like to introduce a non-halakhic perspective on this issue from the Maharal of Prague.^20^ Maharal brilliantly proposed that the *Beit Hamikdosh* (The Temple) represents the heart of *Bnei Israel* (the Israelites), while *limud ha-Torah* (Torah study) represents the brain of *Bnei Israel*. It is not coincidental that Hashem punished us by destroying the *Beit Hamikdosh* because we could spiritually recover. Destroying or taking away our capacity of *limud ha-Torah* would be the irreversible spiritual death of our people. In a tangential way, could the Maharal be favoring brain cessation over cardiac arrest as the definition of halakhic spiritual death?^21^,^22^

Regarding our case, if we follow the view that this woman is halakhically dead, maybe we must remove her from life support because of the obligation to bury the deceased immediately (*k’vod ha-met*)*—*honor of the deceased)^23^ and *nivul ha-met* (desecration of the dead).^24^ If done respectfully, there are at least three exceptions where a deceased body does not have to be buried immediately: (1) for the honor of the deceased, e.g. to obtain a coffin or shrouds;^25^ (2) to gather family or community for burial;^21^ and (3) to assess the legal or medical circumstances about an untimely death.^26^ Thus, medical circumstances that would include saving the life of a fetus would override the obligation to bury the woman immediately. In contemporary practice, reasons for delaying burial include circumstances in which postmortem medical investigation is required. Forensic examinations, undertaken to clarify the cause of death in cases of suspected homicide or malpractice, preserve the deceased’s dignity by preventing false suspicion and are thus permitted by leading decisors such as Rabbi Moshe Feinstein. Similarly, genetic or toxicological studies, when they provide life-saving knowledge for family members or communities, fall under the rubric of *pikuach nefesh* and may even obligate delaying burial. Public health inquiries, such as confirming infectious disease as a cause of death, are also allowed for the same reason.

Interestingly, regarding the permissibility of an autopsy, in Tractate Chullin^27^ the question is discussed whether physicians may perform an autopsy on a murder victim to determine if they were a *treifah* (a person with a fatal, non-curable condition). If they were a *treifah*, the murderer would not be liable for the death penalty, as the person would have died shortly anyway.

Rabbi Moshe Feinstein^28^ and Rabbi Waldenberg^29^ permit autopsies when there is a strong halakhic or medical rationale, especially to save life (*pikuach nefesh*), clarify cause of death, or advance urgent medical knowledge. The Talmud initially raises the question of whether autopsies are permitted, and while the rabbis ultimately forbid it in cases where it is unlikely to arrive at a definite conclusion, the discussion itself implies that if the autopsy had a clear, life-saving purpose, it would be permitted and would override the Halakhah of *nivul ha-met*.^30^,^31^

Rabbi Moshe Sofer (the Chatam Sofer) allows an autopsy if it will help treat a current patient.^32^ Rabbi Yechezkel Landau (the Noda BiYehuda)^31^,^33^ permitted an autopsy on a person who died after a surgical procedure, thus allowing postmortem examination specifically to determine the cause and circumstances of the death. However, he cites a crucial condition: there must be another person in the same community suffering from the same disease who could potentially be saved by the knowledge gained from the autopsy. The Chazon Ish^34^,^35^ expands the leniency of the Noda BiYehuda concerning autopsies performed for the sake of knowledge. While the Noda BiYehuda permitted autopsy only when a specific, endangered patient was immediately present (*choleh le-faneinu*), the Chazon Ish argues that when a widespread, currently active disease or epidemic is the cause of death, the resulting danger is realistic and imminent, making the situation halakhically equivalent to having a sick person before us. Therefore, an autopsy may be performed to gain knowledge vital for saving lives threatened by the spreading disease, as the overriding principle of *pikuach nefesh* supersedes the prohibitions of *nivul ha-met* and *k’vod ha-met*. This leniency applies only to immediate, present dangers, not to remote or speculative future benefits. In the context of autopsies, some modern *poskim* might use the idea metaphorically: since today medical knowledge is globally accessible (via the internet, databases, etc.), there is always effectively a *choleh le-faneinu*, i.e. there is always a patient whose case can benefit from an autopsy or postmortem investigation. The Chazon Ish permits an autopsy if the disease is common and poses a real threat, even without a specific patient present.^36^ Rabbi Asher Weiss^37^ and Rabbi Avraham S. Avraham^38^ note that because medical knowledge today is instantly global, the principle of *pikuach nefesh* can apply even without a specific patient present. They argue that the Noda BiYehuda’s requirement of *choleh le-faneinu* (a sick person before us) may be understood more broadly: if the information gained from an autopsy can immediately benefit patients worldwide, it is as if a patient is “before us.” Rabbi Yechiel Yaakov Weinberg^39^ suggests that in our modern world, with global communication and medical knowledge dissemination, the halakhic category of *choleh le-faneinu* could be greatly expanded—potentially allowing autopsies not only when a patient is physically present, but when it is reasonably likely that the knowledge will save lives elsewhere.

The second halakhic issue in our case of delivering a fetus in a pregnant brain-dead woman is the “*nefesh* status” of an embryo. A basic debate about this issue emerges from the views of Rashi and the Rambam. The core of their disagreement stems from their interpretation of the Talmudic discussion in Mishnah Ohalot^40^ regarding the case of a woman in difficult childbirth.

If a woman is having difficulty in giving birth, one cuts up the fetus within her womb and extracts it limb by limb, because her life takes precedence over that of the fetus. But if the greater part was already born, one may not touch it, for one may not set aside one person’s life for that of another.^40^

Rashi’s view is: *lav nefesh hu* (it is not a soul/person).^41^ However, Rashi continues to explain *yatzah rosho* (if its head has crowned and emerged):

For as long as it has not come out into the world, it is not a *nefesh*, and it is permitted to kill the fetus in order to save its mother. But if its head has crowned, one may not touch it to kill it, for it is considered like one who is born, and we do not set aside one *nefesh* for another *nefesh*.^41^

Rashi’s position is that prior to birth if the fetus poses a threat to the mother’s life, it can be sacrificed to save the mother, because the mother’s life, as a fully recognized *nefesh*, takes precedence over that of the fetus, which is “not a *nefesh*.” Rashi’s view, *lav nefesh hu*, aligns with the literal interpretation of Exodus^42^:

When [two or more] parties fight, and one of them pushes a pregnant woman and a miscarriage results, but no other damage ensues, the one responsible shall be fined according as the woman’s husband may exact, the payment to be based on reckoning.^42^

However, Ramban^43^ infers that the Talmud is specifically referring to a one-day-old-child.

The Sages interpret “no damage” (*ason*) as referring to the mother. If only the fetus is lost, the perpetrator pays a fine (monetary compensation for property damage) and does not incur the death penalty. This distinction implies that the fetus does not have the same legal status as a born person for whom homicide laws apply.

The Talmud in Tractate Niddah (44b) learns from the Torah verse “And he that smites any man mortally, shall be put to death”^44^ that someone who kills a one-day-old baby is liable for his murder. The Talmud explains that the reason for the phrase “any man” in this verse indicates that this ruling applies in any case, even in the case of a one-day-old baby born from a full-term pregnancy. Thus, when the Torah states “any man,” it includes a child who is a single day old. Again, this would seem to imply that if one aborts a fetus, it would not be included in this category.

The Maharal^45^ expands Rashi’s view into a philosophical ontology of life and identity. He argues that the fetus derives its soul and vitality entirely from the mother during gestation. The fetus is not yet a *nefesh bifnei atzma* (independent soul); rather, it exists in potentiality, not actuality. Birth is the ontological moment of soulhood. This supports Rashi’s halakhic view: as long as the fetus has not emerged, it cannot be treated as a separate human being with equal status. In addition, according to the Maharal only birth creates ontological independence.

In contrast, the Rambam’s approach arrives at a similar practical conclusion regarding abortion to save the mother’s life, but through a different halakhic mechanism. He states:

Therefore, the Sages have ruled regarding a pregnant woman who is in difficulty giving birth, that one may cut up the fetus in her womb, whether with a knife or by drugs, because the fetus is like a pursuer—a *rodef* who is attempting to kill her. But if its head has already emerged, one may not touch it, for one does not set aside one life for another.^46^

Rambam introduces the concept of *rodef* (a pursuer) to explain the permissibility of terminating the fetus to save the mother. The law of *rodef* states that if someone is actively pursuing another person with intent to kill, the pursued party (or a third party) may kill the pursuer to save the victim’s life. There are several other Talmudic uses of the term *rodef*. The earliest and most fundamental source for the halakhic concept of rodef is in the Tractate Sanhedrin.^47^ The sages outline the principle that a person who is “pursuing” another to murder or sexually assault them can and must be stopped, even if it requires killing the attacker. Rambam applies this to the fetus: in a life-threatening birth, the fetus is considered a “pursuer” of the mother’s life, and therefore its life can be forfeited to save hers. In his use of the term *rodef*, the Rambam implies that a fetus possesses a status approaching a *nefesh* (personhood), but its life can be sacrificed due to its unwitting “pursuit” of the mother’s life. If the fetus were truly “nothing” or merely a “limb of its mother” in all respects, there would be no need to invoke the *rodef* principle; it would simply be a medical procedure on the mother’s body. By calling it a *rodef*, Rambam implicitly acknowledges the fetus as having a degree of independent life, albeit one that is superseded by the mother’s life due to her immediate danger.

The Mizrachi^48^ critiques Rashi’s position and explicitly defends Rambam’s reasoning. He argues that *ubar yerech imo* (fetus is as limb of its mother) is not absolute, and the fetus can be treated as a quasi-independent entity in halakhic contexts. The Mizrachi accepts the legal fiction of *rodef* to justify fetal termination when the mother’s life is in danger—even before labor. He rejects Rashi’s reduction of the fetus to an appendage and instead emphasizes conflicting halakhic rights. He maintains that the fetus is a quasi-independent entity. The Ha’Meiri^49^ appears to support Rashi’s position as he states: “The fetus is not included in [the category of] nefesh until it emerges into the air of the world.”

There is also an interesting Talmudic *sugya* (Talmudic discussion) suggesting that a fetus has significance as a *nefesh*.^50^ According to the Talmud, the *Shechinah (*Divine Presence of G-d) rests upon the community of Israel under specific circumstances. For example, the text presents a scenario where the presence of a pregnant woman could be a deciding factor for the resting of the *Shechinah* upon Israel, illustrating that the fetus has a legal status that can be counted, and a miscarriage could therefore cause a change in the spiritual status of the community. This is the only Talmudic source to suggest that a fetus has a significant status in the eyes of Torah. Ramban (Nachmanides)^51^ on *makeh ish va-met* (whoever strikes any person mortally shall surely be put to death) explains that the verse excludes a fetus from the legal punishment for murder because the Torah calls *nefesh* only that which has emerged into the air of the world—but adds: “But the fetus has life and a soul [*nefesh*] to grow and to feel.” Here, Ramban affirms that a fetus possesses a *nefesh* in the sense of life-force and sentience, even though halakhically it is not classified as a *nefesh* for capital punishment. Radak^52^ comments on the version *Be-terem etzarkha ba-beten yedaʿtikha* (before I formed you in the womb, I knew you) that this shows Hashem’s knowledge of the person even as an embryo. “He was considered before Him as a complete person, for already the soul was in him.”

Following the opinion of the Rambam, one should maintain life support for a brain-dead pregnant woman until the fetus can be delivered. This is because the fetus, due to its status as a “pursuer” of the mother’s life, is considered to be “almost a *nefesh adam*” (a human life) and is thus worthy of being saved if the mother is no longer alive.

The other halakhic parameter is found in Tractate Arakhin^53^ that states that if a pregnant woman is dying, her fetus always dies before she does. This was understood to mean that the life of a fetus is inextricably linked to the life of the mother; if the mother dies, the fetus cannot survive. This created a halakhic paradox. If a brain-dead person is considered halakhically dead, then the survival of a fetus in their womb would contradict the Talmudic statement that the fetus always dies before the mother.

Rabbi Shlomo Zalman Auerbach realized that there might be a crucial difference between a natural death (as in Talmudic times, where the body’s functions gradually cease) and a modern situation where medical interventions sustain a life. In a natural death, the mother’s cardiovascular system gradually fails, cutting off oxygen and nutrients to the fetus. In contrast, a brain-dead woman on a ventilator has a heart that is still beating and has an intact circulatory system to provide continuous life support to the fetus.

To test this hypothesis and bridge the gap between ancient texts and modern medicine, Rabbi Auerbach proposed an experiment on a pregnant sheep.^54^ Rabbi Professor Avraham Steinberg helped to design the experiment, to create a situation that met the halakhic criteria for death while maintaining the conditions necessary for the fetus to survive. The experiment first involved decapitating a pregnant sheep, which is an unequivocal form of halakhic death.^55^,^56^ The mother sheep was then kept on life support (a ventilator) to maintain its circulatory system, and veterinarians successfully delivered a healthy lamb that lived for several years. The result of the sheep experiment provided the crucial halakhic evidence that Rabbi Auerbach needed. It demonstrated that the Talmudic statement about the fetus dying before the mother referred to the conditions of natural death, not to a situation where the mother’s body is physically dead (as in decapitation) but her life-sustaining functions are artificially maintained.

In summary, we propose that those *poskim* who believe that brain cessation constitutes halakhic death would maintain that it is permitted, and even a religious obligation, to delay burial for months and maintain life support for the brain-dead pregnant woman to allow for the delivery of the baby via C-section.

## CARDIAC DEATH AS THE HALAKHIC ENDPOINT OF LIFE

Many rabbinic authorities, such as Rabbi Mordechai Willig^13^ and Rabbi J. David Bleich,^14^,^57^ maintain that death occurs only upon the irreversible cessation of heartbeat and respiration. These Rabbis offer a different interpretation of the Yoma 85a which discusses rescuing a buried individual on Shabbat. They argue that the text’s focus on checking for breath at the nostrils is not the ultimate definition of death. Instead, they view it as the most reliable and readily observable sign of life available at the time the Talmud was written. They maintain that the true halakhic criterion for death is the cessation of all vital bodily functions, particularly the heartbeat. In addition, Rabbi Bleich uses the Talmudic interpretation of Genesis,^58^ “All in whose nostrils was the breath of the spirit of life,” which is interpreted as the defining sign of life. While breathing is the primary sign, these rabbinical authorities argue that the heartbeat is also a crucial indicator. The heart’s function is the underlying criterion for life, and breathing is merely a more readily observable sign that the heart is still circulating oxygenated blood to the brain and the rest of the body. Therefore, as long as the heart is beating, a person is considered alive, even if they are on a respirator.

Support for the central role of the heart in human physiology also comes from Rabbenu Bahya, who identifies the *lev* (heart) as the king of the body and the central organ governing all limbs and the repository of thought.^59^ The implication is that Rabbenu Bahya might support the Halakhah that the heart’s cessation would signify the end of human life, since the heart is both the biological pump and the spiritual throne of the soul’s powers.

According to those rabbis who define halakhic death as cardiac cessation, a woman whose heart is still beating—even if artificially sustained—is considered halakhically alive and all normal considerations of *pikuach nefesh* apply. Thus, maintaining the life support system on this brain-dead pregnant woman would be mandated. In our case of A.S. there is another halakhic issue. At the time that the physicians can extract the fetus, via C-section, this procedure might constitute a dangerous medical procedure that could result in the final death of the mother.

The risks of surgery, anesthesia, and complications, like hemorrhage, infection, or cardiac arrest, are relevant. Even if she were in a coma and could not consciously feel pain, anesthesia would still be a medical necessity to prevent the body from having a physiological reaction to surgical trauma. The body can still react to pain through involuntary movements, increased heart rate, and other responses. The C-section procedure would add a layer of medical risk and complexity to her already fragile state. The critical halakhic question according to the rabbis who favor cardiac cessation as death is whether physicians can intervene to save the fetus, even if doing so might hasten the mother’s death.

One needs to ask whether a C-section in modern times constitutes a “life-threatening” situation. Could physicians minimize the danger to the brain-dead pregnant woman by inducing vaginal delivery using prostaglandins and oxytocin? In this way physicians may be able to deliver the fetus and continue to maintain the woman’s life on the respirator. Searching the literature there are two documented cases of a brain-dead pregnant woman who delivered a baby via natural birth and not C-section.^60^,^61^ Currently, in most hospitals in the USA, C-section mortality is rare with rates often reported as 2–6 per 100,000 procedures in Caucasian women.^62^,^63^ One could argue that if medical technology today can ensure the stability and not further endanger the medical state of a brain-dead pregnant woman then physicians would be allowed to deliver the fetus via C-section. The Rema^64^ ruled that one should not perform a surgical procedure to extract the fetus, because it is a great danger to the mother. However, today, with advanced surgical techniques and anesthesia, C-sections are generally safe, and many *poskim*—including Rabbi Moshe Feinstein and Rabbi Avraham Steinberg—permit them when medically indicated. The Rema’s ruling is understood as contextual, not an absolute halakhic ban. The fact that A.S. underwent a C-section on June 13 and her medical status was stable until June 17, when the respirator was removed, supports this proposed halakhic decision.

There may be other potential solutions. For example, the respirator could be connected to a Shabbat clock that is designed to turn off the respirator at a specific date. The use of a ventilator connected to a timer would work as follows. The medical staff and/or family would assess the medical status of the patient who is connected to a ventilator with a Shabbat clock. If the patient’s medical condition is improving, then the physicians would press a button to reset the timer and continue the life support. However, if the patient’s medical condition deteriorates before the timer is scheduled to turn off, then the physicians would do nothing and allow the Shabbat clock to automatically turn off the ventilator and allow the patient to die.

The use of a Shabbat clock in our case might circumvent a fundamental principle in Jewish law: the prohibition against actively hastening death. While Jewish law permits and even mandates violating almost all other prohibitions (including those of Shabbat) to save or extend a life, a halakhic distinction is often made between *withholding* life support and *withdrawing* it.^65^ Many halakhic authorities prohibit actively disconnecting a piece of life-sustaining equipment (withdrawing life support), such as a respirator because it actively hastening death. By using a Shabbat timer, the doctor is not actively turning off the machine; he or she is simply not resetting the timer. This is seen by some halakhic authorities as a permissible way to allow the patient to die with dignity while still respecting the prohibition against directly hastening death. This is often framed with the metaphor of not “placing one’s finger on a flickering candle.” This distinction is not universally accepted, and some authorities require utilizing all necessary means to prolong life regardless of prognosis. In one of his responsa, Rabbi Moshe Feinstein considers the obligation to provide a ventilator to a critically ill patient. He states that one is obligated to use all available means to extend life, even for moments, regardless of the patient’s prognosis.^66^

The problem in instituting this Shabbat timer protocol is the question of how long physicians must wait after the respirator has stopped before they declare the patient dead and perform the C-section.^67^ In Israel, physicians have observed that in cases when life support is stopped for 5 mins the hearts of some patients can be revived. However, in patients where the respirator is turned off for 10 minutes, the heart cannot be revived. This creates a major medical and halakhic problem. If one waits 5 minutes before performing the C-section on a brain-dead woman, then the patient is not halakhically dead. If one waits 10 minutes, the patient is halakhically dead but the fetus may not survive the hypoxia. Thus, the use of a Shabbat timer in our case will not solve our halakhic dilemma.

A second potential solution is to halakhically identify the brain-dead pregnant woman (as opposed to the fetus) as a *rodef* and allow the woman to be sacrificed to save the fetus. Post-term pregnancy is traditionally defined as one that continues beyond 42 weeks of gestation. While most pregnancies that go past the due date are healthy, the risk of complications for the fetus begins to increase after 40 weeks, and the risk increases substantially after 41 weeks. The potential harm to the fetus from an extended pregnancy primarily stems from two medical issues (which can even arise before 40 weeks).^68^,^69^ Firstly, the placenta—the organ that provides the fetus with oxygen and nutrients—begins to age and may become less efficient over time. If the pregnancy continues past 40 weeks, the nutrients and oxygen supply to the fetus can be reduced. Secondly, the volume of amniotic fluid can decrease (oligohydramnios), which increases the risk of the umbilical cord being compressed during labor. This compression can restrict blood flow and oxygen to the fetus. In fact, after a 42-week gestation, the pregnancy is officially considered post-term, and the risks of fetal distress, stillbirth, and other complications significantly rise. Thus, after 40 weeks (or maybe even before 40 weeks) of gestation, Halakhah may consider the pregnant woman as a *rodef* to her embryo allowing the physicians to save the embryo over the woman. However, the medical risks and dangers of extending pregnancy are probably not relevant today because medical technology is so sophisticated that the risks to the fetus in post-term pregnancies can be adequately managed and the fetus would not be in a state of *sacanat nefashot* (danger to life). Thus, the idea of the pregnant woman being halakhically classified as a *rodef* would not apply.

A final potential solution is based on the Ramban’s comments to Yoma 82a which states: “A pregnant woman who smells food and feels a craving—if not satisfied, she may be in danger—should be fed until she is calmed.” Ramban rules:

Any doubt of danger to life overrides Shabbat and Yom Kippur, even a remote doubt, and certainly a definite danger. So too, a pregnant woman who smells food—if the fetus is endangered—she is fed until she is calmed.^70^

He explains that even if the fetus is only 40 days old or even less than 40 days old, it is considered sufficiently developed to warrant concern for its life. He aligns with the view that *pikuach nefesh* applies even to potential life, and that Halakhah permits violating Yom Kippur’s fast to protect the fetus. Similarly, in our case we are dealing with a woman who is likely to die within a year, whereas her fetus, if delivered, would live for many years and keep many *Shabbatot*. Thus, the Ramban might rule that the viable fetus will overrule the status of the brain-dead woman. This potential ruling is based on the principle of *pikuach nefesh*—the obligation to preserve life—even when the life in question is potential or not yet fully formed.

While no *posek* explicitly states that a fetus overrides the halakhic status of a brain-dead mother, several authorities draw on Ramban’s commentary to Yoma. They interpret the Ramban^70^ as ruling that if a pregnant woman craves food on Yom Kippur, she must be fed—even when the fetus is less than 40 days old or the danger is only uncertain (*safek sakanat nefashot*). This suggests that the principle of *pikuach nefesh* extends even to potential life.

Rabbi Asher Zelig Weiss argues that maintaining life support for a brain-dead mother to allow a viable fetus to be delivered constitutes a clear case of *pikuach nefesh*.^71^ Similarly, Rabbi Shlomo Zalman Auerbach notes that even very early fetal life activates *pikuach nefesh*, permitting violation of prohibitions.^72^

## CONCLUSION

Judaism teaches responsibility, compassion, and reverence for life. Halakhah responds not with legal rigidity but with ethical pluralism, allowing a spectrum of legitimate positions grounded in deep values. The case in Georgia presents a challenging intersection of medical ethics, legal considerations, and religious beliefs. Jewish law provides a framework for understanding the status of the fetus and the definitions of death, but it also calls for compassion and respect for all lives. In the Georgia courtroom, halakhic principles can provide a framework for analysis—urging us to listen, preserve, and honor the sanctity of life. By examining the Talmudic sources and the perspectives of classical and modern rabbinic authorities, we can navigate these complex issues with a deeper understanding of the halakhot that guide us. In Halakhah, life is not measured solely by the beat of a heart or the hum of a ventilator.

The Torah commands us to choose life, but it also commands us to choose wisely. *Pikuach nefesh* overrides nearly every mitzvah, yet we are also warned not to desecrate the dead, not to hasten death, and not to confuse compassion with convenience. These tensions are not contradictions—they are invitations to moral greatness.
